# Determinants of engaging in sedentary behavior across the lifespan; lessons learned from two systematic reviews conducted within DEDIPAC

**DOI:** 10.1186/s12966-015-0293-2

**Published:** 2015-10-09

**Authors:** Johannes Brug, Mai Chinapaw

**Affiliations:** Department of Epidemiology & Biostatistics, EMGO Institute for Health and Care Research, VU University Medical Center, van der Boechorststraat 7, 1081 BT Amsterdam, The Netherlands; Department of Public and Occupational Health, EMGO Institute for Health and Care Research, VU University Medical Center, van der Boechorststraat 7, 1081 BT Amsterdam, The Netherlands

**Keywords:** Sedentary behavior, Screen time, Determinants, Systematic review

## Abstract

The present commentary provides a brief overview of and reflections on the joint findings of two reviews of the present evidence regarding correlates, predictors and/or determinants of sedentary behavior among youth and older adults published in the International Journal of Behavioral Nutrition & Physical Activity.

In both reviews, the included studies were predominantly conducted in Europe, the US, and Australia. Most studies were limited to TV or ‘screen’ time rather than sedentary behavior and relied on self-report. In both age groups there is a lack of qualitative studies as well as studies looking into the more motivational and contextual potential determinants of sedentary behavior. Both reviews indicate that to date there is limited evidence on the determinants of sedentary behaviour in youth and older adults. In youth, age and weight status were identified as determinants of sedentary behavior, with more sedentary time among older and heavier kids. In adults, age and retirement were determinants, with older and retired elderly sitting more.

## Background

Sedentary behavior refers to any waking behavior characterized by an energy expenditure ≤ 1.5 metabolic equivalent (MET) while in a sitting or reclining posture [1]. It is the state people are in while watching TV, desk work, during most meetings, at school, reading, computer activities, or just hanging out. It is what most people nowadays do for the vast majority of waking hours. In recent years, it has repeatedly been argued that too much –or too much prolonged- sitting may lead to ill health independent of physical activity. Evidence suggests that even when people comply to recommended levels of physical activity, if they are sedentary for much of the rest of the day, they have an increased risk for cardiometabolic ill health and premature death. Among adults quite compelling evidence supports the hypothesis that sedentary behavior is associated with all cause mortality after adjustment for physical activity levels [2]. However, the evidence that too much sitting is detrimental to health in youth is less convincing and inconsistent [3, 4]. This may be due to lack of high quality studies and/or the fact that such effects will emerge only later in life [5]. Furthermore, recent evidence suggests that it is prolonged, uninterrupted sitting that may be most harmful, rather than total sedentary time [6, 7] and children are much more likely to interrupt –albeit often briefly- their sedentary activities much more regularly [5].

If too much (prolonged) sitting is harmful, interventions should be considered to encourage people to reduce and/or interrupt their sitting time. Besides, reducing sedentary behavior may contribute towards the promotion of physical activity, for which an abundance of evidence for the health-enhancing effects exists. To inform such interventions, insights are needed in the individual and contextual determinants, predictors and correlates of sedentary behaviors, i.e. the reasons and conditions that make people sit too much, for too long.

Within the Determinants of Diet and Physical Activity (DEDIPAC) joint action of the European Joint Programming Initiative ‘A Healthy Diet for a Healthy Life [8], a series of systematic and umbrella reviews has been or is being conducted within three thematic areas, i.e. regarding measurement of dietary, physical activity and sedentary behaviors; regarding policy and multilevel interventions to promote healthy behaviors; and to provide an overview of the evidence-base regarding potential determinants for behaviors that have not been reviewed recently. The latter was the case for determinants of sedentary behaviors, and three systematic reviews have been conducted to review the present evidence regarding correlates, predictors and/or determinants of sedentary behavior across the life span, i.e. among youth, adults and older adults. Two of these reviews –of research conducted among youth and older adults- are published in the International Journal of Behavioral Nutrition & Physical Activity [9, 10] and in the present commentary we provide a brief overview of and reflections on the joint findings.

These two reviews focus on two different age groups: youth and older adults. Both reviews used a social-ecological framework to guide the review analyses, thus recognizing the importance of both individual, social and contextual level determinants. Both reviews were conducted according to established review protocols that were made available in PROSPERO, and used the same established methodology to assess and take into account the quality of the studies included in the review.

Stierlin and colleagues reviewed 37 studies among youth. Because of the abundance of available studies in this age group, they were able to restrict their review to longitudinal–intervention and observational- studies only. Their results show that at the individual level, age and weight status were associated with sedentary behavior, with more total sedentary time as well as screen time among older and more screen time among heavier kids. Chastin and colleagues focused on older adults and included 22 studies in their review, almost all cross-sectional, observational studies. Their results for example show that older and retired elderly sit more. Both reviews reveal a lack of studies on true motivational and contextual potential determinants of sedentary behavior in these age groups.

First of all, the fact that separate reviews were conducted for different age groups makes perfect sense: it seems logical that determinants or correlates of sedentary behavior are different for youth than for older people. Furthermore, both reviews show that the number of studies exploring or testing presumed determinants of sedentary behavior is growing rapidly; most of the studies included in the reviews are from recent dates. However, the number of available studies among youth was much larger than among adults and elderly. Stierlin and colleagues were therefore able to restrict their review to longitudinal studies only, i.e. to studies with a much more rigorous design to identify predictors and determinants, rather than ‘just’ correlates of sedentary behavior. This wealth of studies among youth makes sense on the one hand but seems illogical on the other. If one argues that we should start early in life to discourage sedentariness, we should also start with gaining more and better insights into the determinants of sedentary behaviors among youth. However, because the evidence for the ill health effects of sedentary behavior is much stronger among adults -including the elderly- it would make more sense to focus determinant research more on adult and elderly populations.

Across both reviews it is evident that exploring potential determinants of sedentary behavior is predominantly based on studies conducted in Europe, the US, and Australia. Studies conducted among populations in other countries and regions of the world are largely lacking and such research in other regions should urgently be encouraged and supported.

Many studies included in the review do not focus so much on sedentary behavior in general, but specifically on TV or ‘screen’ time as the dependent variable. However, TV and screen time may only make up a small proportion of total sedentary time. For example, Verloigne et al. found that among 10–12 year olds total self-reported screen time (a summation of TV and computer time) was significantly associated with total sedentary time (as measured with accelerometers) but explained only 7 % of variance [11]. Moreover, there is a lack of studies exploring determinants of prolonged uninterrupted sitting.

The fact that most studies included in the reviews rely on self-report measures, is another limitation and clearly recognized by the authors; self reported sedentary time grossly underestimates total sedentary time. Moreover, existing self-report measures do not reflect the nowadays popular habit of “media multitasking” among youth, which may have specific determinants in itself. Accelerometers, preferably including inclinometer functionality so that anatomical position (e.g., lying down, sitting, standing) is also identified, are typically regarded as the preferred standard for measuring sedentary time. However, accelerometers also have disadvantages. The objective raw data need to be translated into sedentary time. This translation (data-reduction) is based on a number of subjective decisions that varies between studies [12]. Likewise, different definitions of what constitutes sedentary time or a sedentary bout are used. Consensus and consistency in data-reduction procedures as well as definitions is needed to bring the sedentary behavior research field further [13]. Furthermore, accelerometer data do not provide information about the type of sedentary behavior people engage in nor of the context where sedentary activities take place. Only three studies in older adults and none among youth used an inclinometer [9].

The ‘determinants’ identified in the different reviews generally say more about who engages more or less in sedentary behavior than *why* they do so. Information about the association of such variables as age, weight status, employment status et cetera with sedentary behavior is interesting and relevant for deciding who may be in most need for interventions and policies to discourage sedentariness, i.e. for targeting the interventions towards the most sedentary population groups. However, to inform the content of interventions we need to know about the motivational and contextual reasons for engaging in sedentary behaviors, i.e. insights in important and modifiable mediators of behavioral change. The reviewed studies do not provide much evidence regarding such mediators and further research focusing specifically on motivation, abilities and opportunities as well as unconscious processes that may induce and sustain changes in sedentary behavior is essential. Many studies were not specifically designed for exploring determinants of sedentary behavior. As a result, the usual suspects have been explored (e.g. age, gender, weight status, SES) and not potential determinants that are specific for sedentary behavior.

Many of the reviewed studies as well as the reviews themselves, state that a social-ecological framework was applied to explore potential determinants. Such frameworks posit, however, that different determinants influence health behavior at different levels, i.e. some determinants are more distal and may influence behavior mediated or moderated by more proximal or upstream determinants. Such potential mediation or moderating pathways between different levels of determinants were not explored in the studies included in the reviews, and such research is warranted.

Finally, qualitative studies exploring possible determinants of sedentary behavior are also largely lacking. Both reviews mainly include studies exploring researcher-identified determinants of sedentary behavior. We completely agree with the conclusion in the older adults review that: “future research should integrate the views and opinions of older subjects themselves in a systems based approach of health promotion through the life course” [10]. This may be even more important for youth as adult researchers may be less able to understand and anticipate the reasons and conditions for sedentary activities among children and adolescents. Active involvement of the target groups may not only shed new light on modifiable determinants of various sedentary behaviors in specific subgroups but also result in acceptable and thus more effective interventions.

## Conclusions

Both reviews indicate that to date there is limited evidence on the determinants of sedentary behaviour in youth and older adults. In youth, age and weight status were identified as determinants of sedentary behavior, with more sedentary time among older and heavier kids. In adults, age and retirement were determinants, with older and retired elderly sitting more. In both age groups there is a lack of studies looking into the more motivational and contextual potential determinants of sedentary behavior.

## Abbreviations

SES: Socio-economic status
